# Supplementary material for: Divergent roles of HDAC1 and HDAC2 in the regulation of epidermal development and tumorigenesis
Source: EMBO J. 2013 Nov 15;32(24):3176–91. doi: 10.1038/emboj.2013.243 (PMC3981143; doi:10.1038/emboj.2013.243)

SUPPLEMENTARY INFORMATION – WINTER, MOSER et al.

SUPPLEMENTARY MATERIAL
SUPPLEMENTARY METHODS
SUPPLEMENTARY FIGURES (FIG. S1-S7)
SUPPLEMENTARY TABLES (PROVIDED AS EXCEL FILES: SUPPLEMENTARY TABLE 1-3)

SUPPLEMENTARY MATERIAL

PRIMER

Genotyping primer

HDAC1-flox (5’ GGTAGTTCACAGCATAGTACTT 3’ and 5’ CCTGTGTCATTAGAATCTACTT 3’),
HDAC2-flox (5’ GGTAGTTCACAGCATAGTACTT 3’ and 5’ GTTACGTCAATGACATCGTCTT 3’),
K5-Cre (5’ TAATCGCCATCTTCCAGCAG 3’ and 5’ CAATTTACTGACCGTACAC 3’),
K5-SOS-F (5’ TTCCGCCTCTCCGGGCTTACAGC 3’ and 5’ GAGAGATAAAGGGTTTCTTCGCTTCC 3’),
*Waved2 (*5’ ATAACCTGACACTTGTCAGAGTAC 3’ and 5’ TTTGCAATCTGCACACACCAGTTG 3’);

pPCR primer for mRNA expression:

Ada (5’ GTTGTGCGTGAGGCTGTG 3’ and 5’ GTCGTCTGTGTTGAGTGAGTAG 3’),
Epgn (5’ TGAGCGAAGAAGCAGAGG 3’ and 5’ CCACAGCATACGAAGTTAGG 3’),
CD34 (5’ CCTGATGAACCGTCGCAGTTGGAG 3’ and 5’ GGGTCACATTGGCCTTTCCCTGAGT 3’),
Claudin3 (5’ GACGTAGTCCTTGCGGTCGTA 3’ and 5’ GCGGCTCTGCTCACCTTAGT 3’),
Claudin 8 (5’ TCAGAATGCAGTGCAAGGTC 3’ and 5’ AGCCGGTGATGAAGAAGATG 3’),
c-Myc (5’ AGTGTTCTCTGCCTCTGC 3’ and 5’ CTGCTGTTGCTGGTGATAG 3’),
Hdac1 (5’ GCCTCTTCCACGCCATCG 3’ and 5’ AAGCAGCAGACGGACATCG 3’),
Hdac2 (5’ CCCTTTAGGTGTGAGTACAT 3’ and 5’ AACCTGGAGAGGACAGCAAA 3’),
Hprt (5’ GCTGGTGAAAAGGACCTCT 3’ and 5’ CACAGGACTAGAACACCTGC 3’),
Hoxc13 (5’ ACTGGGCTCTTTCCAATGG 3’ and 5’ GCTCACTTCGGGCTGTAG 3’),
keratin15 (5’ TGGACATCAAGACTCGGCTGGAAC 3’ and 5’ ACCGCTGCTTCCTTCTCTGACACC 3’),
Klk6 (5’ GCCCGTGTTTGAAGGACTC 3’) and (5’ TCTGTTTGCCGTAGGTTGTG 3’),
Lgr5 (5’ GGGCCTTCAGGTCTTCCTCAAAGTCA 3’ and 5’ CCAATGGAATAAAGACGACGGCAACA 3’),
Lgr6 (5’ GCTGGACCTGACTGACAAC 3’ and 5’ GGTGCCTCCTCTTCCTCTG 3’),
Lhx2 (5’ TCACCTCAACTGCTTCAC 3’ and 5’ GCATCGTTCTCGTTACAG 3’),
MTA1 (5’ TTGTGGATAAGCAGATTG 3’ and 5’ GAGATGTCGTAGATGTTC 3’),
MTA2 (5’ CCTGTTGGATTTATTGGAAG 3’ and 5’ TGGCATTGGCATTGATAG 3’),
Msx2 (5’ GGAGCACCGTGGATACAG 3’ and 5’ TTCCAGTTCCGCCTCTTG 3’),
Occludin (5’ TTGAAGTCCACCTCCTTACAGA 3’ and 5’ CCGGATAAAAAGAGTACGCTGG 3’),
S100a3 (5’ GCAGCAGTGTGAGGATGAC 3’ and 5’ TCGTGGCAGTAGAGACAGAG 3’),
Sin3a (5’ CCGCCATAGCAGCAGTTCATAGC 3’ and 5’ CCAGGAGGCAAGAAGGTGTTAAAGC 3’),
Sox9 (5’ CTCTCCTAATGCTATCTTCAAG 3’ and 5’ GCTCAGTTCACCGATGTC 3’),
Sprr2h (5’TTCCATAGCAACACCTCCATC 3’ and 5’ ACCTCTTCCTTCTGAGCATCTG 3’),
Sprr2i (5’ ATTCAGCACTATGTCTTAC 3’ and 5’ TCTTCTTCTCTTGGTCTC 3’),
Green african monkey beta-actin (5’ GCTGCCCTGAGGCTCTCTT 3’ and 5’ TGATGGAGTTGAAGGTAGTTTCATG 3’);

qPCR primer for ChIP analysis
Ada (-359) (5' GTCGCCGCTAAATCTCCC 3' and 5' ACTCCAATGTCCTCACCTG 3'),
Epgn (-342) (5' ATTATCTTGTCTGCTGTTCTG 3' and 5' TCATCTGGCTGTTCTTACC 3'),
Hoxc13 (5' TTCCTTCAGTTCAGTTAAATGATTCC 3' and 5' CGGGCGA TGGTCACACTC 3'),
Klk6 (720) (5' GGGGTCACTGTGTTCCAGAG 3' and 5' TCTCAGAGTCTCCCAGCTCC 3'),
Lce3b (-165) (5' CCTGGTTCCTGGTTCAATCTTC 3' and 5' TTATCCTGTGCAGTTGCTGATG 3'),
Msx2 (-271) (5' GCAGATTTCCAACATTCTCAGG 3' and 5' GCAGAGTTGTTATTAGGCGATCC 3'),
S100a8 (5' TCCAACACTCCCACTTCCTCAG 3' and 5' AGACAACGCTCTTCCCTTCCAG 3'),
S100A9 (-200) (5' GTTGGATGGAAGGGAAGTG 3' and 5' GGCAGCTCCTTGTTAGATAC 3'),
Sprr2h (-208) (5' GGATGGTCTCTGAGTCACTGC 3' and 5' CCCGCCCTGTAGGTTGTAAAG 3'),
Vstm2l (5' AAGGCTCTGTGACCATTCTG 3' and 5' GAACCAGGCTCATCCAGTC 3');

***In situ* hybridization primer:**
Lgr5 (5' CTCCAACCTCAGCGTCTTC '3 and 5' AAGGCGTAGTCTGCTATGTG '3),
Lgr6 (5' CTGGACCTGACTGACAAC '3 and 5' CGGAGAGTAACACGATGG '3);

ANTIBODIES
Primary antibodies used for Co-immunoprecipitation:
HDAC1 (1:1000; Millipore), HDAC2 (1:1000; Millipore), Sin3A (1:250; sc-994X, Santa Cruz), CoREST (1:1000; 07-455, Upstate), MTA2 (1:2000; M7569, Sigma).
Primary antibodies for immunoblotting:
HDAC1 and HDAC2 (1:1000; Millipore), Sin3A (1:250; sc-994, Santa Cruz), CoREST (1:1000; 07-455, Upstate), MTA2 (1:2000; M7569, Sigma), Keratin 10 (1:1000, Covance), c-Myc (1:250; sc-764, Santa Cruz), Actin (1:20000; A5316, Sigma), Lamin B (1:1000, sc-6216, Santa Cruz), Skp2 (1:500, cs-4313, Cell Signaling).
Primary antibodies for immunoblotting – histone blots: H3 C-terminal (1:500000, ab1791, Abcam), H3K14ac (1:200000, 07-353, Millipore), H3K27ac (1:40000, ab4729, Abcam), H3K4ac (1:20000, 39381, Active Motif), H3K56ac (1:5000, ab76307, Abcam), H4K8ac (1:20000, Sat198, homemade Seiser Lab).
Primary antibodies used for ChIP:
HDAC1 (affinity purified polyclonal serum (Bartl et al, 1997)), HDAC2 (4µg, Abcam ab12169), Sin3A (4µg, sc-994X, Santa Cruz), H3K9ac (4µg, 06-942, Millipore), H4ac (4µg, 06-866, Millipore) and corresponding IgG (Invitrogen) as a control.
Primary antibodies used for IHC:
Ki67 (1:500, NovoCastra), Keratin 1, Keratin 6, Keratin 14, Involucrin, (1:1000, Covance), HDAC1, HDAC2 (1:100, Millipore), BrdU (1:200; 03-3940, Zymed), CD34 (1:50; ab81518, Abcam), GATA3 (1:100; sc-268, Santa Cruz), c-Myc (1:500; sc-769 Santa Cruz)
Primary antibodies used for FACS:
MHC-II PE (1:1000, Biolegend), CD45 APC-Cy7 (1:100, Biolegend), CD11b PE-Cy7 (1:100, Biolegend), F4/80 APC (1:50, eBioscience), Ly6G PE (1:50, Biolegend), 7-AAD PerCP (2.5µL/sample, Sigma).

SUPPLEMENTARY METHODS

Oil O red staining of sebaceous glands

For SG isolation tail skin was incubated in MEM/Dispase solution (5mg/ml) over night at 4°C. Epidermis was separated from dermis, fixed in 4% PFA and lipids were stained with 2mg/ml Oil Red O solution (Sigma) for 10min. Tissues were counterstained with hematoxylin and mounted with glycerin.

Quantification of IHC stainings

Microscopy and quantification analysis

For the quantification of caspase-3, p53 and BrdU positive cells ImageJ software was used. For the quantification of the epidermal thickness, area, Oil Red O, Keratin 6 and Ki67 the Zeiss axiovision software was used.

Quantification of caspase-3, p53, Ki67 and Keratin 6.

For the quantification of caspase-3, p53 and Ki67 positive cells in the IFE positive cells of the basal and suprabasal layers of the IFE were counted and set in relation to the total cell number.

For the quantification of Caspase-3 and p53 positive cells in the HF all positive cells were counted and set in relation to the cell number per HFs.

For the quantification of keratin 6 in IFE the area of keratin 6 expressing cells was measured and set in relation to the length of the basal layer.

Quantification of 5-bromo-2'-deoxyuridine (BrdU)

P5 mice were injected with 50µg/g BrdU 2h prior to sacrifice. For the quantification of BrdU positive cells in the basal and suprabasal layer of the IFE were counted and set in relation to the length of the basal layer. For the quantification of proliferating cells in HFs BrdU positive cells in the HF bulb were counted and set in relation to the number of HFs.

For the quantification of BrdU label retaining cells P6 old mice were injected with BrdU for 72h (pulse). After 25 days chase (P34) BrdU and CD34 IHC was performed. BrdU positive cells (control mice = 128, *Hdac1*$^{\Delta/\Delta ep}$*Hdac2*$^{\Delta/+ep}$ = 165) were counted and categorized according to their localization in IFE, HF or co-localization with CD34.

Quantification of epidermal thickness, HF units and SG size

For the quantification of the IFE thickness the epidermal area and basal layer length were measured. The average epidermal thickness was obtained by dividing the area through the length of the basal layer.

For the quantification of the sebaceous gland size the area of Oil Red O positive cells per HF unit was measured.

For the quantification of hair follicle units tail epidermis was isolated and stained like previously described (Oil O red staining of sebaceous glands). The amount of HF units was counted and set in relation to the epidermal area.

In situ hybridization

For *in situ* hybridization mouse back skin was fixed in 4% PFA, cryopreserved in sucrose and embedded in OCT (Tissue Tek). *In situ* hybridization was performed as previously described (Murko et al, 2010). Labelled probes were generated with the MaxiScript kit (Ambion) and digoxigenin-UTP (Roche). Primers can be found in the Suppl. Information.

Wound healing assay

To address the role of HDAC1 and HDAC2 in wound healing, punch wounds (5mm in diameter) were placed with a sterile punching device (kai medical) at the shaved back of adult (>P35) mice. Wounds were monitored by photography and measured with a sliding caliper each day for two weeks. Skin biopsies were isolated 14 days after injury and used for histology.

Dye exclusion assay

Newborn (P0) pups were killed by CO_2 inhalation and incubated for 1min in 25, 50, and 75% methanol in PBS, followed by a 1min incubation in 100% methanol, and a descending series of incubations in 75, 50, and 25% methanol in PBS for 1min. Pups were washed in PBS and stained with 0.1% toluidine blue O (Sigma) for 1 in. Destaining was done in PBS.

Trans-epidermal water loss (TEWL) measurements

Except for P5 pups, mice were shaved with an animal trimmer and TEWL was measured with a Tewameter form Courage and Khazaka, model TM300 (Cologne, Germany). Measurements were made at constant humidity and room temperature and were carried out in triplets per mouse.

FACS analysis

Dermis and epidermis from P5 (backskin) and adult mouse (ear sheets) were separated after overnight incubation in dispase solution (see above) at 4°C. Epidermis was cut into small pieces and incubated for 30min at 37°C in 0.2mg/ml DNAse (Sigma) in RPMI. Dermis was cut and treated with RPMI containing DNAse and 1.6mg/ml collagenase IV (Worthington) for 30min at 37°C. A single cell suspension was prepared by passing the dissociated tissue through a 70µm cell strainer. Cells were washed with FACS buffer (1% BSA, 0.1% NaN_3, PBS) and unspecific antibody binding was blocked with anti-mouse CD16/CD32

(eBioscience). Approximately 1x10E6 cells per tube were stained with respective antibodies for 30min at 4°C. 7AAD (Sigma) was added shortly before measurement for dead cell discrimination. Data were acquired on an FACS-Aria and analyzed with FlowJo software. For antibodies used for FACS analysis see Antibody list above.

Histone isolation

For histone isolation epidermis was homogenized in histone lysis buffer (10mM Tris-HCl pH6.5, 50mM sodium disulfite, 10mM $MgCl_2$, 10mM sodium butyrate, 8.6% sucrose, 1% TritonX-100) and histone isolation was performed as previously described (Lagger et al, 2002). Equal amounts of histone extracts were separated by SDS–PAGE and immunoblots were done as descibed above. Histone lysis buffer was supplemented with protease inhibitor cocktail (cOmplete, Roche) and aprotinin (Sigma). For antibodies see Suppl. Information above.

Cycloheximide protein stability assays

Primary keratinocytes were treated for different periods of time with 0.36mM cycloheximide (Sigma). Whole cell extracts were prepared and the decline in protein levels was analyzed on immunoblots.

Cycloheximide protein stability assays after transient transfection

COS-7 cells were grown in 6-well dishes and transfected at 80% confluence with mouse c-Myc expression plasmid (generous gift from S.B. McMahon) with TurboFect Transfection Reagent (Thermo Scientific). 24h after transfection the medium was changed and cells were treated with solvent (DMSO) or 2µM MS-275 in DMSO. After 2 hours 0.36mM cycloheximide was added for 30min to 4 hours. Cells were collected in Hunt buffer (20mM Tris-HCl pH8.0, 100mM sodium chloride, 1mM EDTA, 0.5% NP-40, supplemented with inhibitors- as described above) and proteins were analyzed on immunoblots.

REFERENCES

Bartl S, Taplick J, Lagger G, Khier H, Kuchler K, Seiser C (1997) Identification of mouse histone deacetylase 1 as a growth factor-inducible gene. *Mol Cell Biol* **17:** 5033-5043

Lagger G, O'Carroll D, Rembold M, Khier H, Tischler J, Weitzer G, Schuettengruber B, Hauser C, Brunmeir R, Jenuwein T, Seiser C (2002) Essential function of histone deacetylase 1 in proliferation control and CDK inhibitor repression. *Embo J* **21:** 2672-2681.

Murko C, Lagger S, Steiner M, Seiser C, Schoefer C, Pusch O (2010) Expression of class I histone deacetylases during chick and mouse development. *Int J Dev Biol* **54:** 1527-1537

SUPPLEMENTARY FIGURES

Supplementary Figure S1 (A and B) Overlapping expression of HDAC1 and HDAC2 in the epidermis and in the hair follicle. Fluorescent IHC analysis of HDAC1, HDAC2, K1 and K14 in back skin sections (P5) **(A)** and HDAC1 and HDAC2 in hair follicles **(B)** from adult (P35) control, *Hdac1*$^{\Delta/\Delta ep}$ and *Hdac2*$^{\Delta/\Delta ep}$ mice. Nuclei are counterstained with DAPI. Scale bar: 50µm. **(C)** Mice with deletion of HDAC1 or HDAC2 in the murine epidermis show no obvious phenotype. Pictures of adult control, *Hdac1*$^{\Delta/\Delta ep}$, *Hdac2*$^{\Delta/\Delta ep}$ and *Hdac1*$^{\Delta/+ep}$ *Hdac2*$^{\Delta/+ep}$ mice. **(D)** H&E staining of back skin sections from control, *Hdac1*$^{\Delta/\Delta ep}$ and *Hdac2*$^{\Delta/\Delta ep}$ mice in the second anagen phase (P35). The nuclei were counterstained with Mayer's hemalaun. Scale bar: 200µm. **(E and F)** Quantification of Ki67 positive cells **(E)** and epidermal thickness **(F)** in the back skin of control, *Hdac1*$^{\Delta/\Delta ep}$ and *Hdac2*$^{\Delta/\Delta ep}$ mice. n=3. p=0.8170, 0.5359, 0.3131 and 0.8049. **(G)** Formation of tail scars in *Hdac1*$^{\Delta/\Delta ep}$ (P660) **(H)** IHC labeling of Ki67 in tail skin section of control, *Hdac1*$^{\Delta/\Delta ep}$ and *Hdac2*$^{\Delta/\Delta ep}$ mice (left panel, P180). Quantification of Ki67 positive cells in tail skin sections from control, *Hdac1*$^{\Delta/\Delta ep}$ and *Hdac1*$^{\Delta/\Delta ep}$ scar mice (right panel). n=3, p=0.0004 and 0.0001. scale bar: 50µm **(I)** Relative mRNA expression of *Hdac1* and *Hdac2* in the epidermis of *Hdac1*$^{\Delta/\Delta ep}$ (upper panel) and *Hdac2*$^{\Delta/\Delta ep}$ (lower panel) and their corresponding wildtype littermates. **(***Hdac1*$^{\Delta/\Delta ep}$: n=4; *Hdac2*$^{\Delta/\Delta ep}$: n=3, p=0.0007, 0.3217, 0.7254 and 0.0001). **(J)** Immunoblot analysis of epidermal protein extracts from control, *Hdac1*$^{\Delta/\Delta ep}$ and *Hdac2*$^{\Delta/\Delta ep}$ mice. Membranes were probed with antibodies against HDAC1, HDAC2, and β-actin was used as loading control. Immunoblot signals of HDAC1 and HDAC2 were quantified by densitometric scanning and are shown relative to the β-actin signal. n=4. **(K)** Total HDAC activity was measured in epidermal protein extracts from control, Hdac1$^{\Delta/\Delta ep}$ and Hdac2$^{\Delta/\Delta ep}$ mice and is shown in %. n=3, p=0.1806 and 0.6984. **(L)** Distribution of genotypes 10 days after birth: *Hdac1*$^{f/+}$ *Hdac2*$^{f/f}$ (n=18), *Hdac1*$^{f/f}$ *Hdac2*$^{f/f}$ (n=13), *Hdac1*$^{\Delta/+ep}$ *Hdac2*$^{\Delta/\Delta ep}$ (n=12), *Hdac1*$^{\Delta/\Delta ep}$ *Hdac2*$^{\Delta/\Delta ep}$ (n=0). **(E,F,I-K)** Error bars display s.d.

Supplementary Figure S2 (A) IF staining of HDAC1 and HDAC2 in control, *Hdac1*$^{\Delta/\Delta ep}$ *Hdac2*$^{\Delta/+ep}$ and *Hdac1*$^{\Delta/+ep}$ *Hdac2*$^{\Delta/\Delta ep}$ mice (P130). Nuclei are counterstained with DAPI. Scale bar: 50µm. **(B)** Relative mRNA expression of *Hdac1* and *Hdac2* in the epidermis of *Hdac1*$^{\Delta/\Delta ep}$ *Hdac2*$^{\Delta/+ep}$ and *Hdac1*$^{\Delta/+ep}$ *Hdac2*$^{\Delta/\Delta ep}$ mice and their corresponding wildtype littermates. n=3, p=0.0227, 0.0701, 0.0530 and 0.0231. **(C)** Immunoblot analysis of histone acetylation in the epidermis from control, *Hdac1*$^{\Delta/\Delta ep}$ *Hdac2*$^{\Delta/+ep}$ and *Hdac1*$^{\Delta/+ep}$ *Hdac2*$^{\Delta/\Delta ep}$ mice (left panel). Antibodies against following histone modifications were used: H3K4ac,

H3K14ac, H3K27ac, H3K56Ac and H4K8Ac. An antibody specific for the C-terminus of histone 3 (H3) was used as loading control. Quantification of the immunoblot signals. n=2 to 3 (right panel). **(D)** Quantification of HDAC1 and HDAC2 protein levels in wildtype keratinocytes by calibration of monoclonal and polyclonal HDAC1 and HDAC2 with recombinant proteins and detection and quantification with the Odyssey detection system. Monoclonal antibodies: HDAC1 (10E2 homemade Seiser lab), HDAC2 (3F3 homemade Seiser lab), polyclonal antibodies: HDAC1 (Millipore), HDAC2 (Abcam ab-7029). n=3. **(B-D)** Data are mean ± s.d.

Supplementary Figure S3 Increased signals of p53, caspase 3 and keratin 6 in *Hdac1*$^{\Delta/\Delta ep}$ *Hdac2*$^{\Delta/+ep}$ epidermis. **(A)** Quantification of the number of hair follicle units in control and *Hdac1*$^{\Delta/\Delta ep}$ *Hdac2*$^{\Delta/+ep}$ littermates. n=3, p=1.000. **(B)** IF staining of p53 (upper panel) and caspase 3 (lower panel) in control and *Hdac1*$^{\Delta/\Delta ep}$ *Hdac2*$^{\Delta/+ep}$ hair follicles at P10. scale bar: 50µm. **(C)** IFE of control and *Hdac1*$^{\Delta/\Delta ep}$ *Hdac2*$^{\Delta/+ep}$ mice stained for p53 and caspase 3 at P10. scale bar: 50µm. **(D)** IF staining of BrdU in the IFE (upper panel) and in HF (lower panel) in control and *Hdac1*$^{\Delta/\Delta ep}$ *Hdac2*$^{\Delta/+ep}$ mice (P5) after 2h BrdU labeling. Scale bar: 100µm. (A-C: nuclei were counterstained with DAPI). **(E)** IHC analysis of keratin 6 (K6) in the IFE of control and *Hdac1*$^{\Delta/\Delta ep}$ *Hdac2*$^{\Delta/+ep}$ back skin sections (P30). Scale bar: 100µm. Quantification of K6 positive areas is shown on the right. Error bars indicate s.d. n=3. p=0.0015.

Supplementary Figure S4 *Hdac1*$^{\Delta/\Delta ep}$ *Hdac2*$^{\Delta/+ep}$ mice do not display skin barrier defects or immune cell infiltration. **(A)** TEWL measured at the indicated time points for control and *Hdac1*$^{\Delta/\Delta ep}$ *Hdac2*$^{\Delta/+ep}$ littermates. n=3 to 8. p=0.5574, 0.0832, 0.1006, 0.4400 and 0.7008. **(B)** Expression of tight junction genes in control and *Hdac1*$^{\Delta/\Delta ep}$ *Hdac2*$^{\Delta/+ep}$ mice as shown by qPCR. n=3, p= 0.2175, 0.0541 and 0.1142. **(C)** Toluidine blue staining of *Hdac1*$^{\Delta/\Delta ep}$ *Hdac2*$^{\Delta/+ep}$ mice and control littermates at P0. Successful staining is indicated by the dark blue staining of the navel. **(D-E)** Flow cytometric analysis of percentages of the indicated cell types in epidermal **(D)** and dermal **(E)** cell suspensions isolated from P5 wildtype and *Hdac1*$^{\Delta/\Delta ep}$ *Hdac2*$^{\Delta/+ep}$ mice. **(D,E)** CD45+ cells are shown as percent of viable cells. Error bars indicate s.d. n=3, p=0.7299, 0.6866, 0.04846, 0.5975 and 0.1520.

Supplementary Figure S5 **(A)** *In situ* hybribization with Lgr5-specific riboprobe on skin sections from control and *Hdac1*$^{\Delta/\Delta ep}$ *Hdac2*$^{\Delta/+ep}$ mice at P35. Dashed lines marks HF. Scale bar: 100µm. **(B)** Wound healing assay with adult (P≥300) *Hdac1*$^{\Delta/\Delta ep}$ *Hdac2*$^{\Delta/+ep}$ and control littermates. Photographs of full thickness punch wounds in mice of the indicated genotype is shown every second day after wounding (upper panel) and wound area measured in mm^2 is depicted in the lower panel. Error bars indicate s.d. n=3 to 4. P0-5: p>0.05; p=0.0441,

0.0119, 0.0119, 0.0004, 0.0007, P11-P14: P=0.0001. **(C)** H&E staining of wound sections of control and two different *Hdac1*$^{\Delta/\Delta ep}$ *Hdac2*$^{\Delta/+ep}$ mice two weeks after wounding. Scale bar 1000µm. **(D)** H&E staining (left panel) and IF staining with a c-Myc antibody (right panel) of spontaneously appearing *Hdac1*$^{\Delta/\Delta ep}$ *Hdac2*$^{\Delta/+ep}$ skin tumors of control and *Hdac1*$^{\Delta/\Delta ep}$ *Hdac2*$^{\Delta/+ep}$ mice (P≥300). Wildtype littermate skin was shown as control. Right panel: Nuclei were counterstained with DAPI. Scale bar: 1000, 100 and 50µm.

Supplementary Figure S6 (A) Relative mRNA expression of *Sin3A*, *MTA2* and *MTA1* in control and *Hdac1*$^{\Delta/\Delta ep}$ *Hdac2*$^{\Delta/+ep}$ mice. n=3, p=0.3648, 0.5498 and 0.8384. **(B)** Protein stability assay by cycloheximide treatment of *Hdac1*$^{\Delta/\Delta ep}$ *Hdac2*$^{\Delta/+ep}$ and *Hdac1*$^{f/f}$ *Hdac2*$^{f/+}$ keratinocytes as controls. Immunoblot analysis was performed with antibodies specific for Sin3A and MTA2 and relative intensities of signals were quantified by densitometric scanning and are shown relative to β-actin. The value at time 0 was set to 100%. n=2. **(C)** Expression of *c-Myc* mRNA in control and *Hdac1*$^{\Delta/\Delta ep}$ *Hdac2*$^{\Delta/+ep}$ epidermis shown by qPCR. n=3, p=0.1049. **(D)** COS-7 cells were transiently transfected with an expression plasmid encoding murine c-Myc and treated with the vehicle DMSO or MS-275. Untransfected cells were used as control. First panel: RNA expression of the cells upon c-Myc transfection. Error bars indicate s.d. n=1. Second panel: Immunoblot analysis with an antibody against c-Myc; Lamin B was used as loading control. Relative intensities of signals were quantified by densitometric scanning and are shown relative to Lamin B. The signal for DMSO-treated cells was set to 1. n=2; Third Panel: Protein stability assay by cycloheximide treatment of transfected COS-7 cells in the presence of DMSO or MS-275. Immunoblot analysis with an antibody against c-Myc; Lamin B was used as loading control. Relative intensities of signals were quantified by densitometric scanning and are shown relative to Lamin B. The value at time 0 was set to 1. n=3. **(A-D)** Mean value with s.d. is shown.

Supplementary Figure S7 ChIP analysis of negatively and positively regulated Sin3A/HDAC1/HDAC2 target genes. **(A)** Negatively regulated target genes. Chromatin from littermate controls, *Hdac1*$^{\Delta/\Delta ep}$ and *Hdac1*$^{\Delta/\Delta ep}$ *Hdac*2$^{\Delta/+ep}$ epidermis was immunoprecipitated with antibodies specific for HDAC1, HDAC2, Sin3A, histone H3K9ac, pan-histone H4ac or IgG as control. Precipitated DNA was analyzed by qRT-PCR with primers specific for *S100a8*, *Klk6*, *Ada* and *Vstm2l* (control) promoter regions. Data are representative for at least two independent experiments. Error bars indicate s.d. n=2 to 5. **(B)** Positively regulated target genes. Chromatin from littermate controls and *Hdac1*$^{\Delta/\Delta ep}$ *Hdac*2$^{\Delta/+ep}$ epidermis was immunoprecipitated with antibodies specific for HDAC1, HDAC2, histone H3K9ac, pan-histone H4ac or IgG as control. Error bars indicate s.d. n=3 to 5.

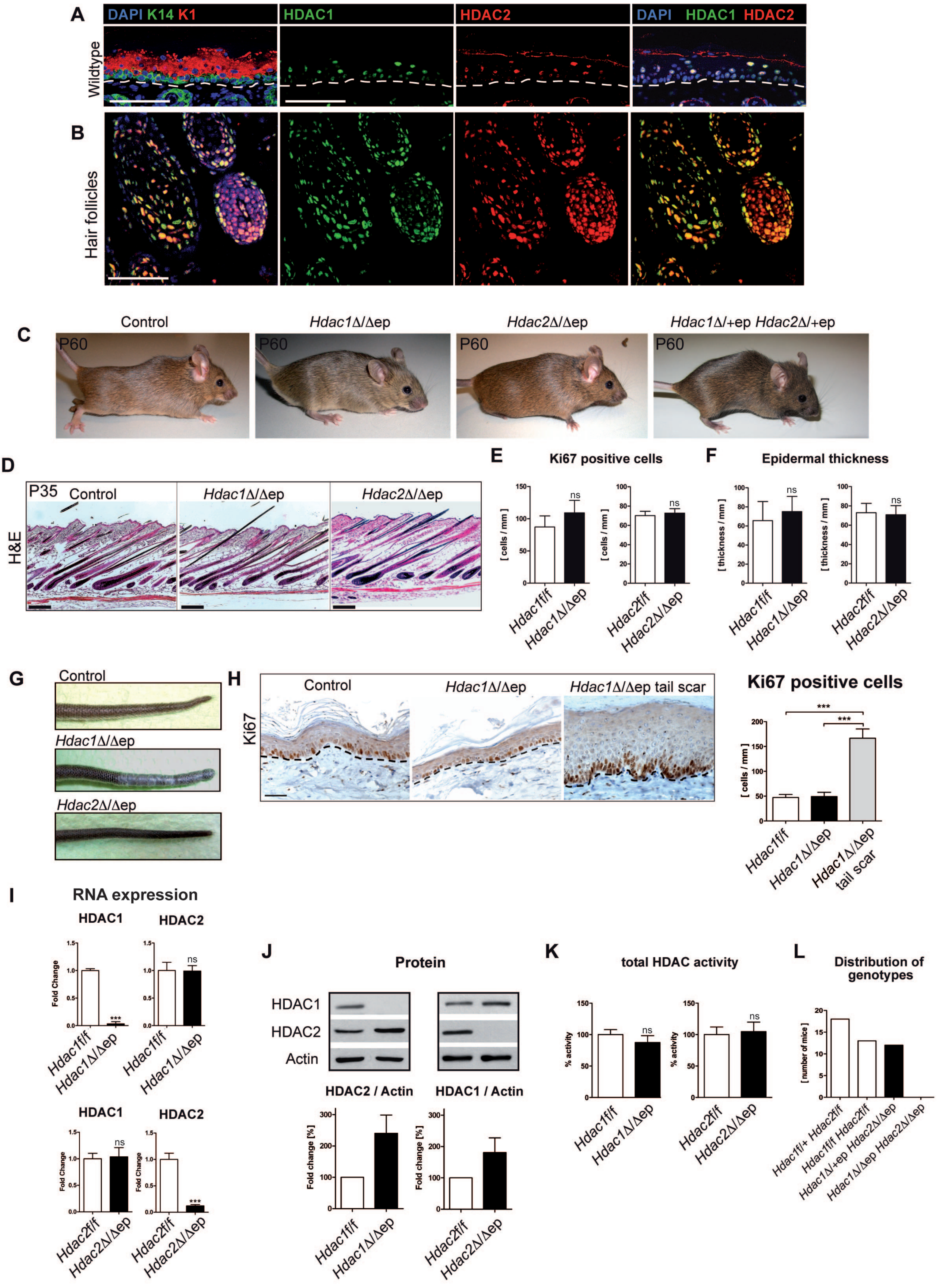
A
DAPI K14 K1
HDAC1
HDAC2
DAPI HDAC1 HDAC2
Wildtype
B
Hair follicles
C
Control
Hdac1Δ/Δep
Hdac2Δ/Δep
Hdac1Δ/+ep Hdac2Δ/+ep
P60
D
P35 Control
Hdac1Δ/Δep
Hdac2Δ/Δep
H&E
E
Ki67 positive cells
[cells / mm]
Hdac1f/f
Hdac1Δ/Δep
Hdac2f/f
Hdac2Δ/Δep
ns
F
Epidermal thickness
[thickness / mm]
G
Control
Hdac1Δ/Δep
Hdac2Δ/Δep
H
Control
Hdac1Δ/Δep
Hdac1Δ/Δep tail scar
Ki67
Ki67 positive cells
***
Hdac1Δ/Δep tail scar
I
RNA expression
HDAC1
HDAC2
Fold Change
J
Protein
HDAC1
HDAC2
Actin
HDAC2 / Actin
HDAC1 / Actin
Fold change [%]
K
total HDAC activity
% activity
L
Distribution of genotypes
[number of mice]
Hdac1f/+ Hdac2f/f
Hdac1f/f Hdac2f/f
Hdac1Δ/+ep Hdac2Δ/Δep
Hdac1Δ/Δep Hdac2Δ/Δep

Winter, Moser Figure S2

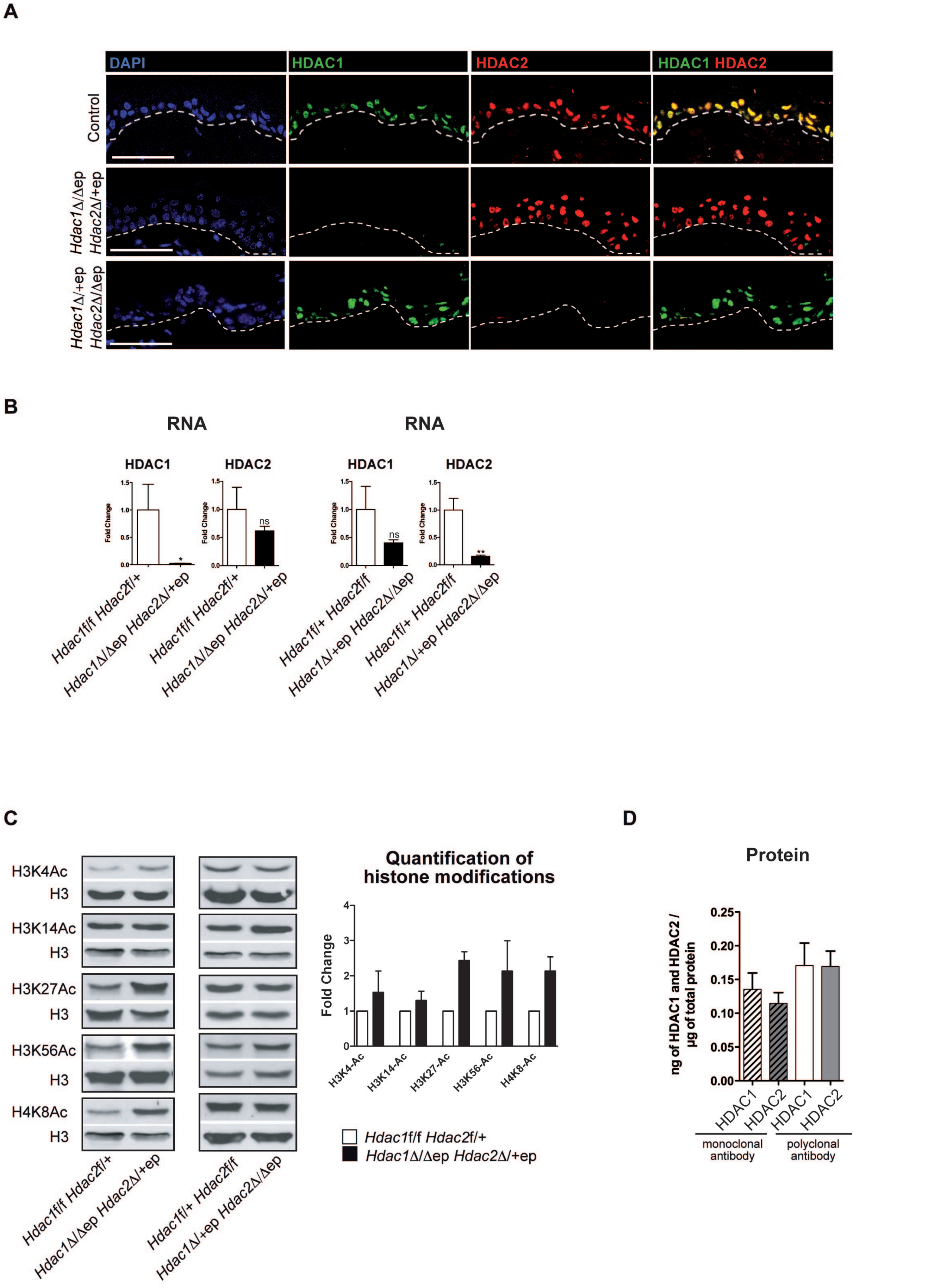

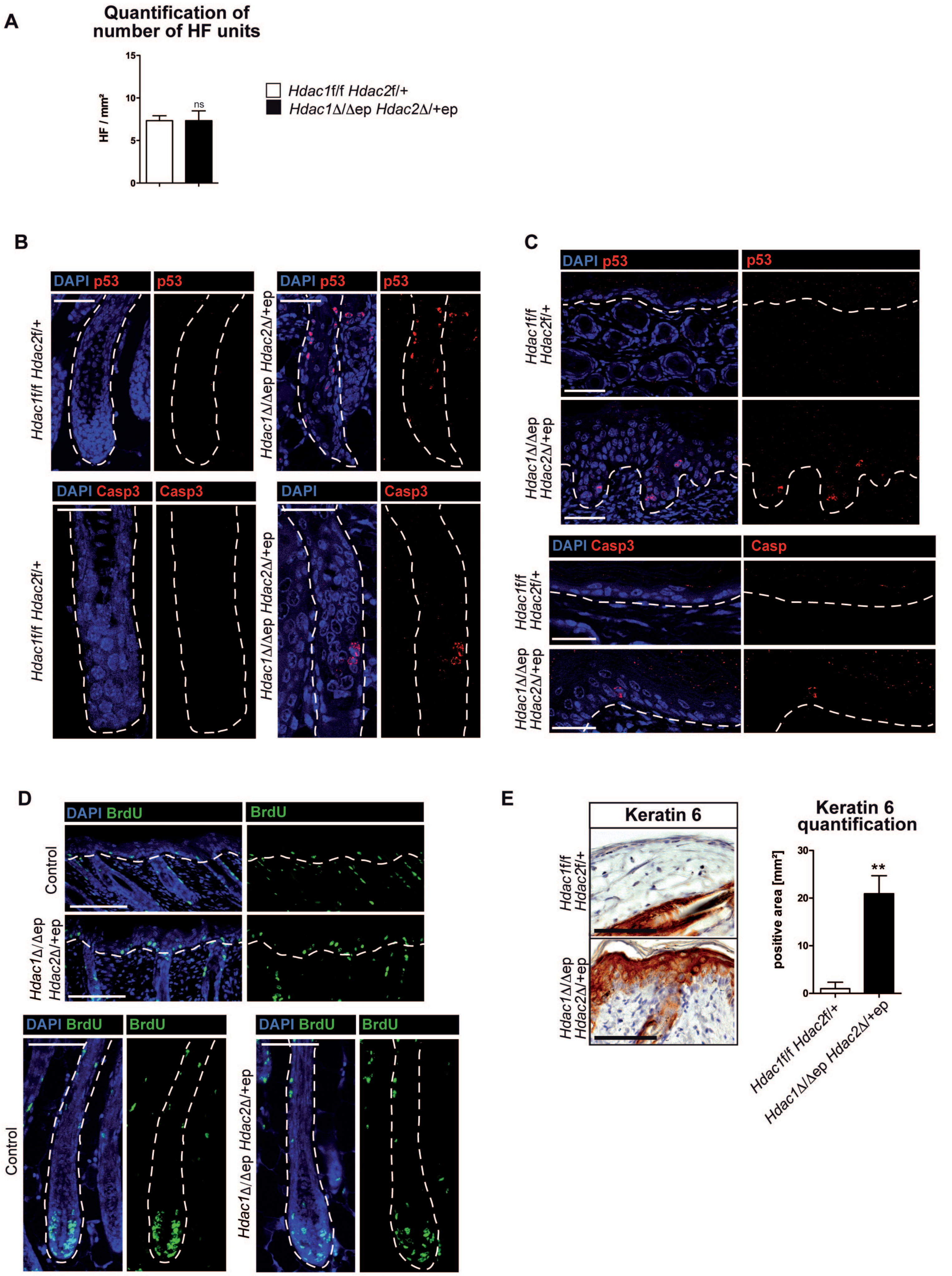
A
Quantification of number of HF units
HF / mm²
15
10
5
0
ns
Hdac1f/f Hdac2f/+
Hdac1Δ/Δep Hdac2Δ/+ep
B
DAPI p53
p53
Hdac1f/f Hdac2f/+
Hdac1Δ/Δep Hdac2Δ/+ep
DAPI Casp3
Casp3
DAPI
C
Hdac1f/f Hdac2f/+
Hdac1Δ/Δep Hdac2Δ/+ep
Casp
D
DAPI BrdU
BrdU
Control
Hdac1Δ/Δep Hdac2Δ/+ep
E
Keratin 6
Keratin 6 quantification
positive area [mm²]
30
20
10
0
**
Hdac1f/f Hdac2f/+
Hdac1Δ/Δep Hdac2Δ/+ep

Winter, Moser Figure S4

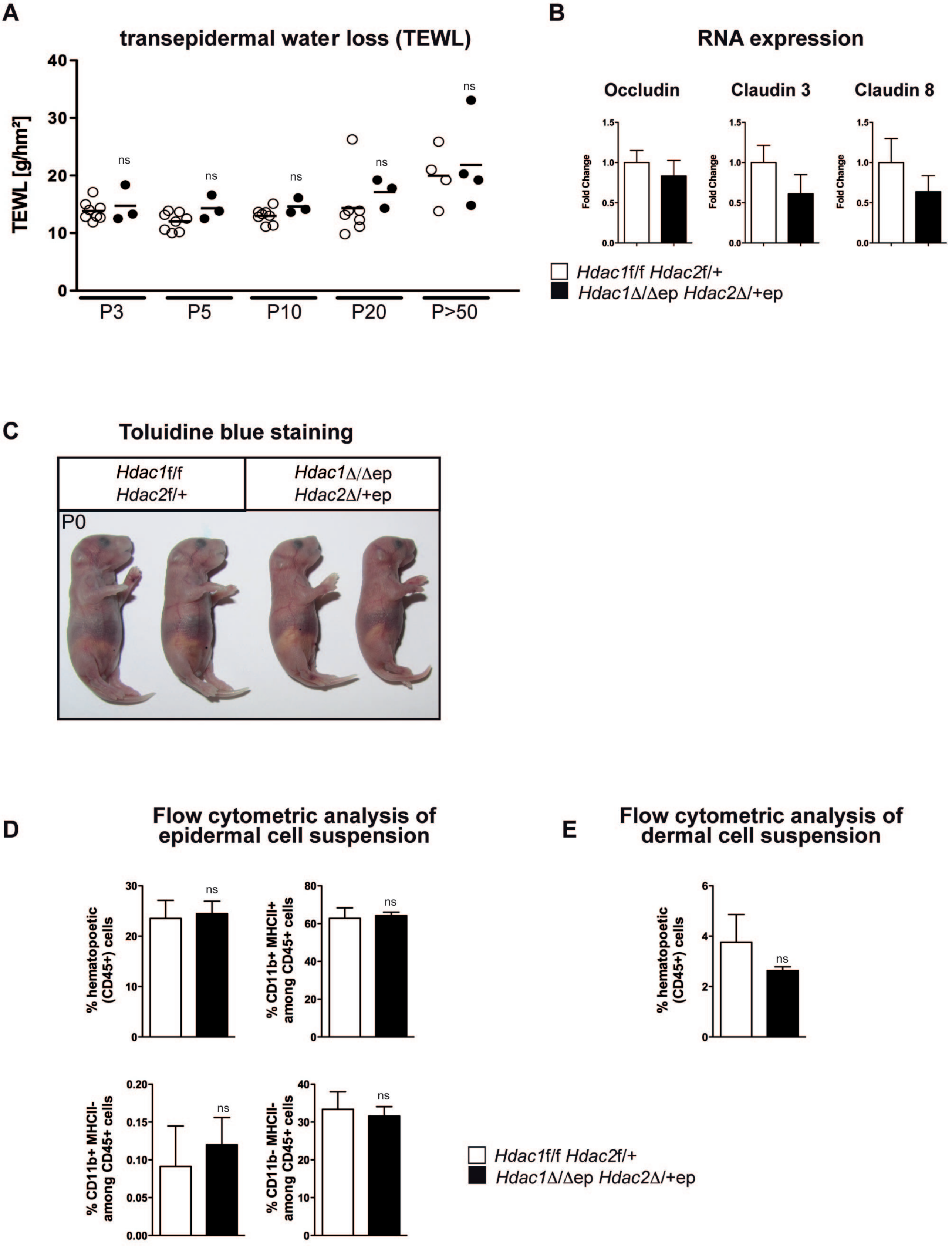

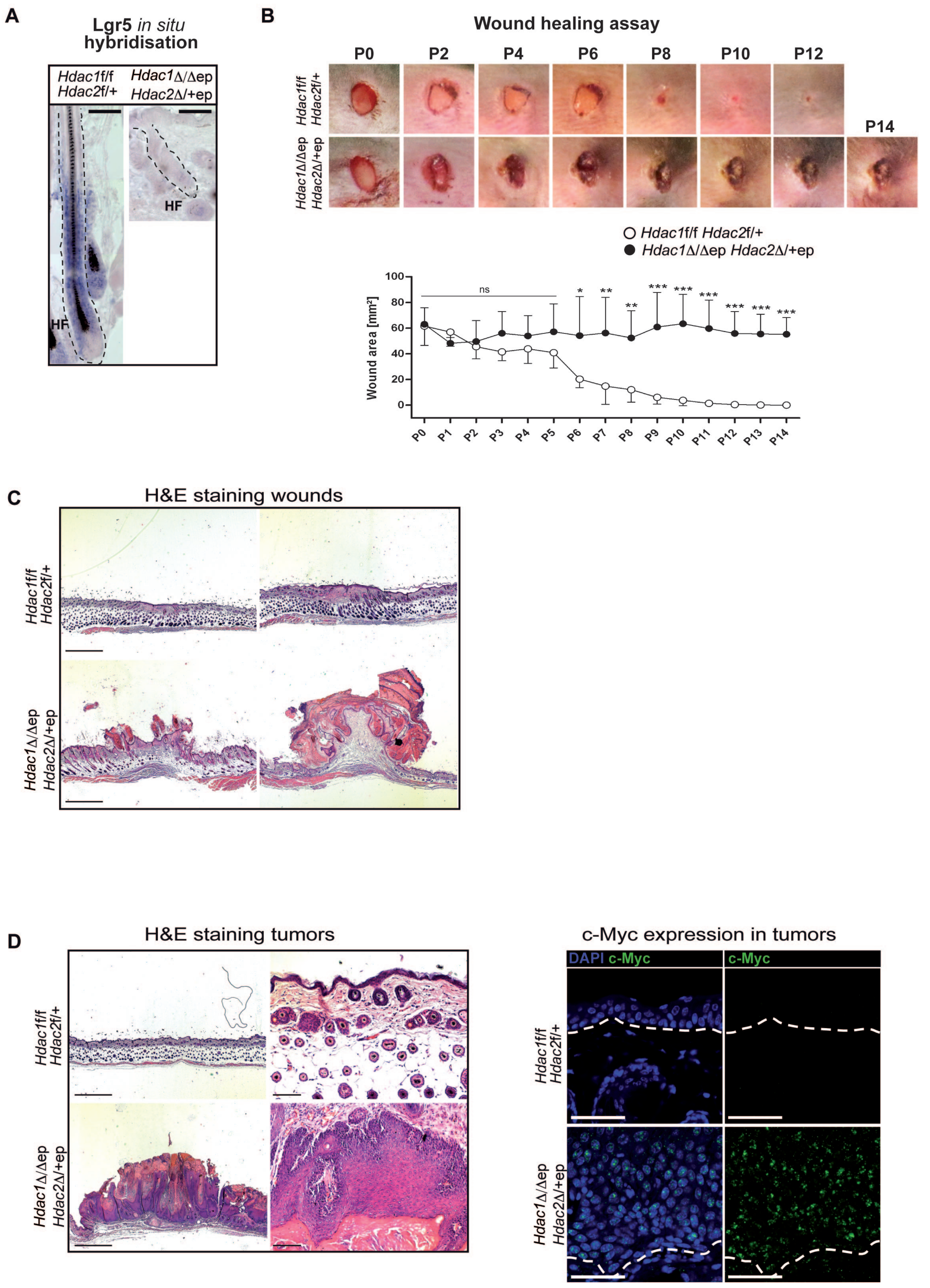
A
Lgr5 in situ hybridisation
Hdac1f/f Hdac2f/+
Hdac1Δ/Δep Hdac2Δ/+ep
HF
HF
B
Wound healing assay
P0
P2
P4
P6
P8
P10
P12
P14
Hdac1f/f Hdac2f/+
Hdac1Δ/Δep Hdac2Δ/+ep
Hdac1f/f Hdac2f/+
Hdac1Δ/Δep Hdac2Δ/+ep
Wound area [mm²]
100
80
60
40
20
0
ns
*
**
**
***
***
***
***
***
***
P0
P1
P2
P3
P4
P5
P6
P7
P8
P9
P10
P11
P12
P13
P14
C
H&E staining wounds
Hdac1f/f Hdac2f/+
Hdac1Δ/Δep Hdac2Δ/+ep
D
H&E staining tumors
Hdac1f/f Hdac2f/+
Hdac1Δ/Δep Hdac2Δ/+ep
c-Myc expression in tumors
DAPI c-Myc
c-Myc
Hdac1f/f Hdac2f/+
Hdac1Δ/Δep Hdac2Δ/+ep

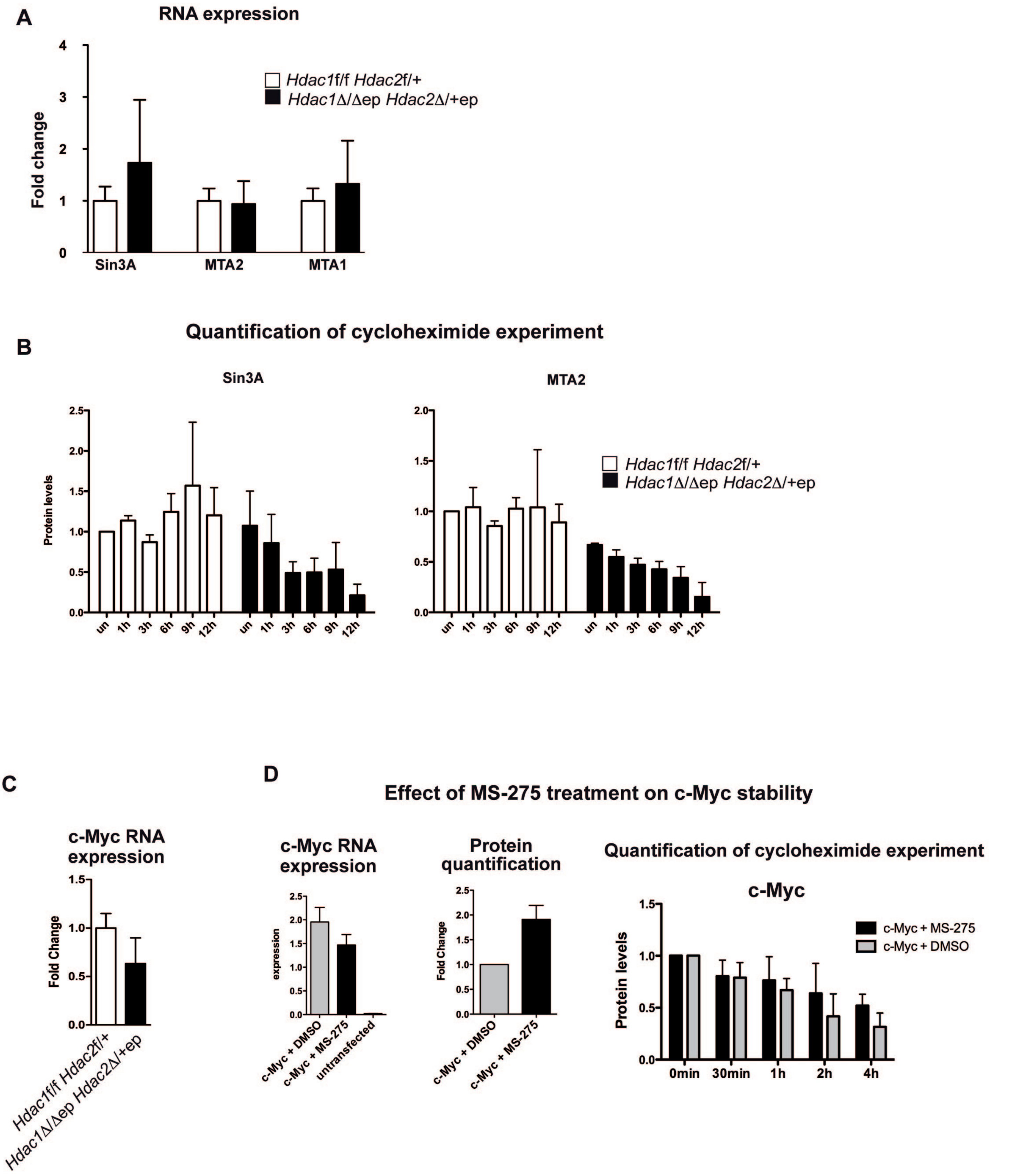
A
RNA expression
Fold change
Hdac1f/f Hdac2f/+
Hdac1Δ/Δep Hdac2Δ/+ep
Sin3A
MTA2
MTA1
B
Quantification of cycloheximide experiment
Sin3A
MTA2
Protein levels
un
1h
3h
6h
9h
12h
C
c-Myc RNA expression
Fold Change
D
Effect of MS-275 treatment on c-Myc stability
c-Myc RNA expression
expression
c-Myc + DMSO
c-Myc + MS-275
untransfected
Protein quantification
Quantification of cycloheximide experiment
c-Myc
Protein levels
c-Myc + MS-275
c-Myc + DMSO
0min
30min
1h
2h
4h

Winter, Moser Figure S7

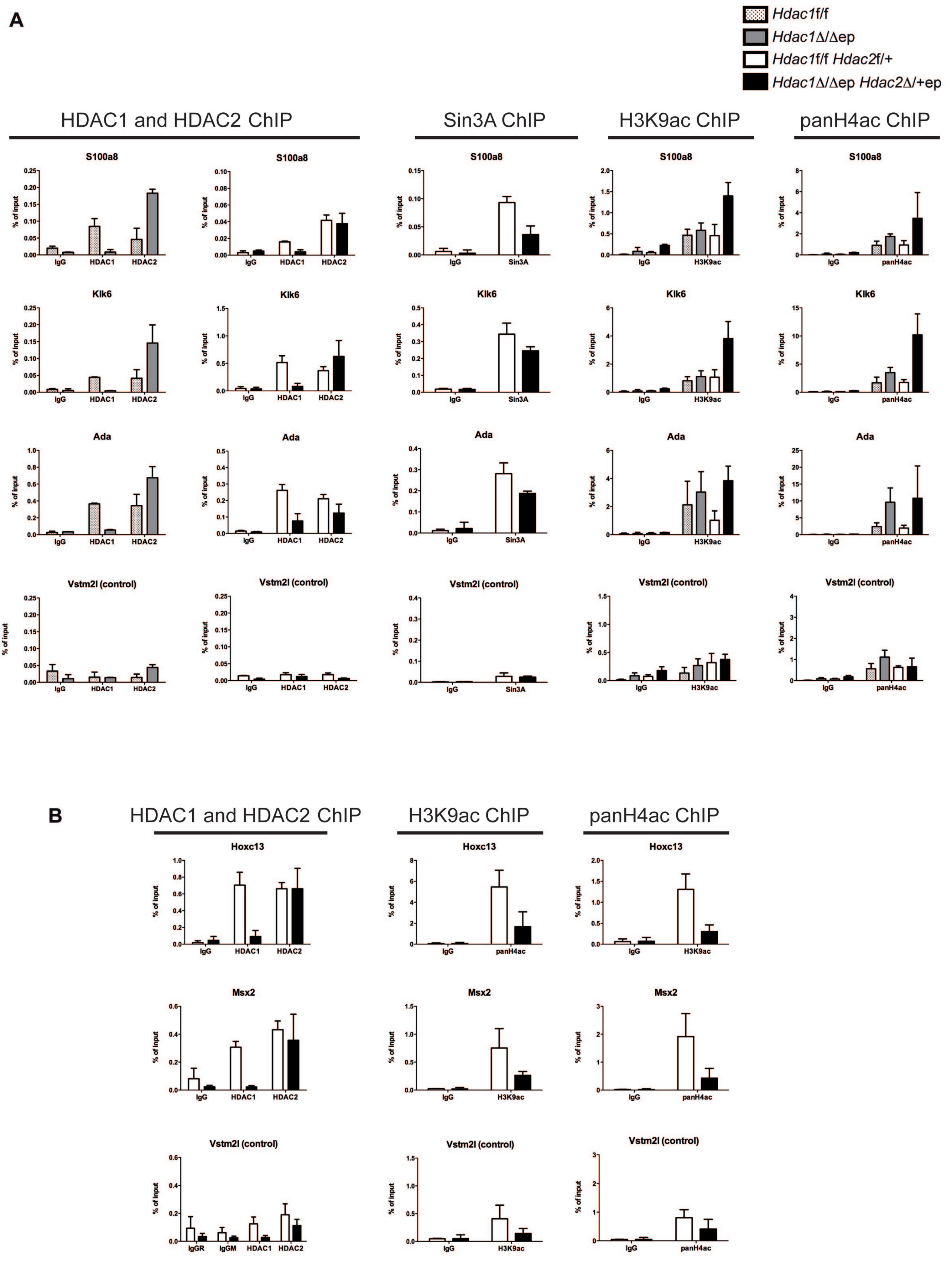

Supplement: Supplementary Information [file emboj2013243s1.pdf]
