# Supplementary material for: Divergent roles of HDAC1 and HDAC2 in the regulation of epidermal development and tumorigenesis
Source: EMBO J. 2013 Nov 15;32(24):3176–91. doi: 10.1038/emboj.2013.243 (PMC3981143; doi:10.1038/emboj.2013.243)
Supplement: Supplementary Table legends [file emboj2013243s5.doc]

**Supplementary TABLEs (PROVIDED AS EXCEL FILES)**

**Supplementary Table 1.** Deregulation of genes associated with hair formation, hair follicle development and the hair cycle in the epidermis of *Hdac1*^∆/∆ep^*Hdac2*^∆/+ep^ mice (p>0.05, at least 2-fold change in expression).

**Supplementary Table 2.** Deregulated genes in the epidermis of adult *Hdac1*^∆/∆ep^ (HD1 ko) and *Hdac1*^∆/∆ep^ *Hdac2*^∆/+ep^ (HD2s) mice (p>0.05, at least 2-fold change in expression). Up-regulated genes (UP) and down-regulated genes (DOWN) are listed separately.

**Supplementary Table 3.** Gene ontology analysis of genes up-regulated in the *Hdac1*^∆/∆ep^ *Hdac2*^∆/+ep^ epidermis using DAVID software (<http://david.abcc.ncifcrf.gov/>).
