# Supplementary figures and images for: Divergent roles of HDAC1 and HDAC2 in the regulation of epidermal development and tumorigenesis
Source: EMBO J. 2013 Nov 15;32(24):3176–91. doi: 10.1038/emboj.2013.243 (PMC3981143; doi:10.1038/emboj.2013.243)

Fig 1

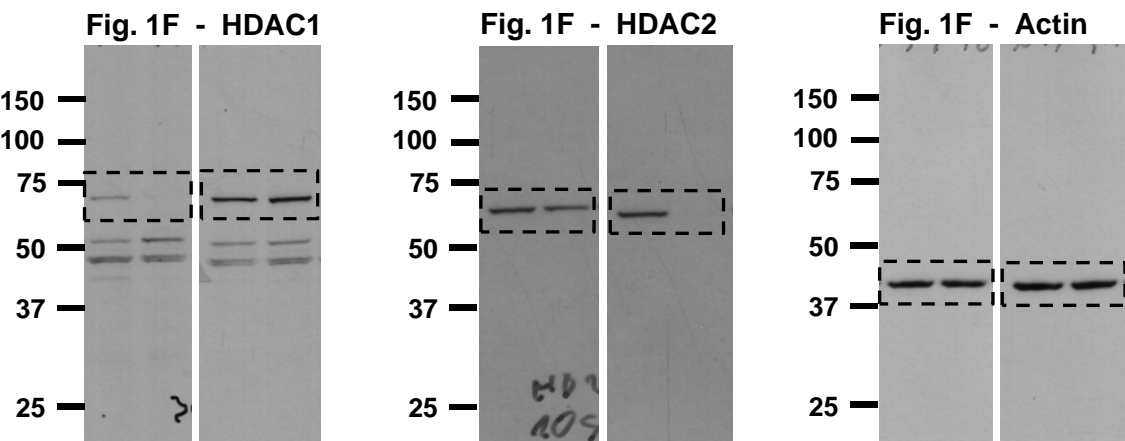

Supplement: Source Data for Figure 1 [file emboj2013243df1.pdf]

**Fig 4**

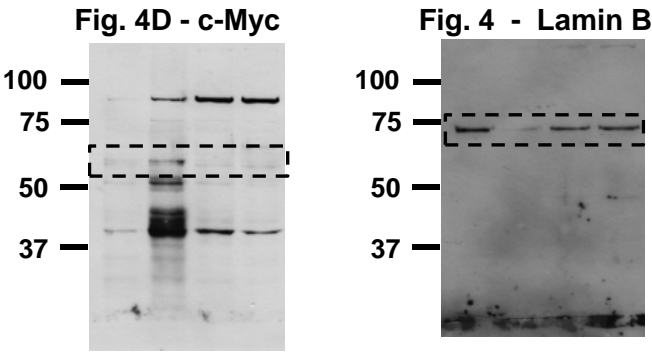

Supplement: Source Data for Figure 4 [file emboj2013243df4.pdf]

**Fig 7**

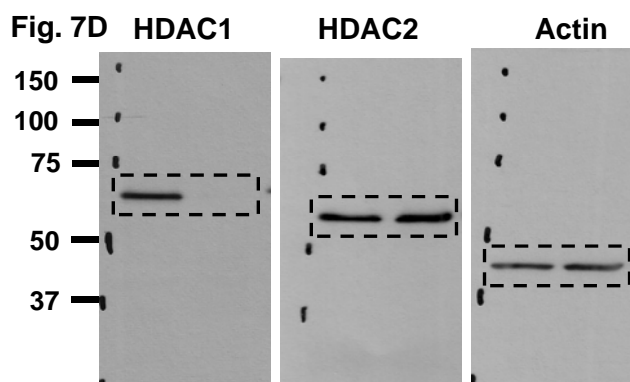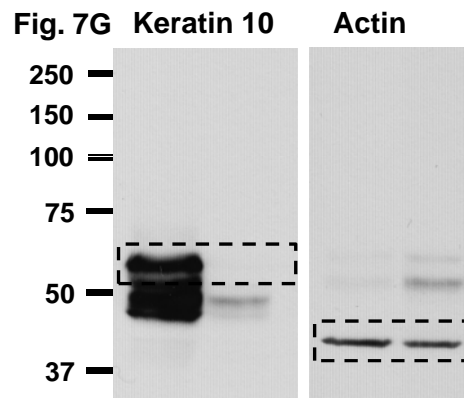

**Fig. 7I** Input

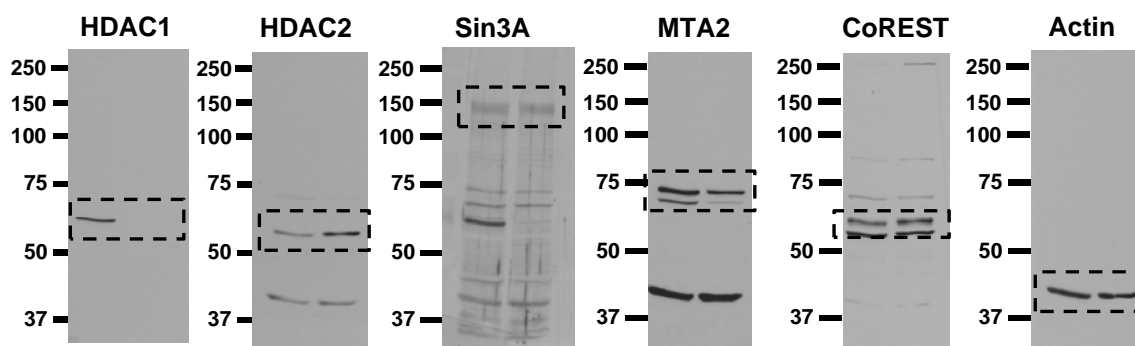

**Fig. 7I Sin3A-IP**

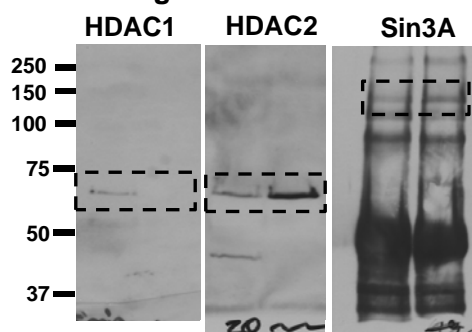

**Fig. 7I MTA2-IP**

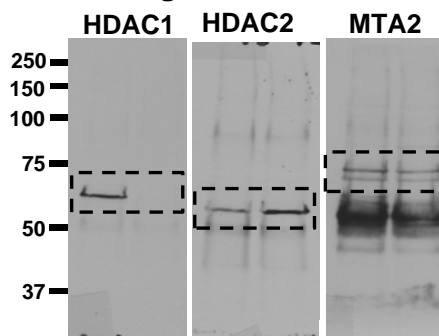

**Fig. 7I CoREST**

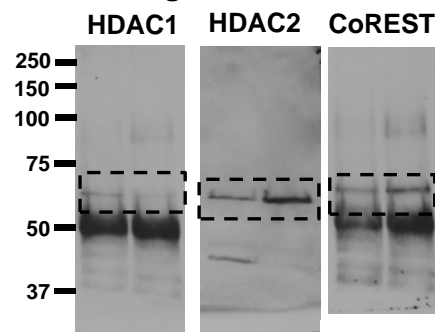

**Fig. 7K**

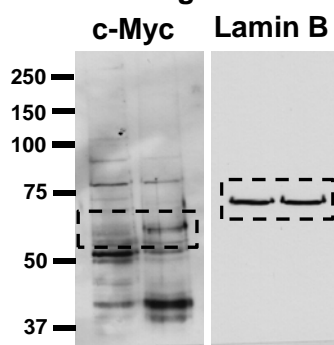

**Fig. 7K**

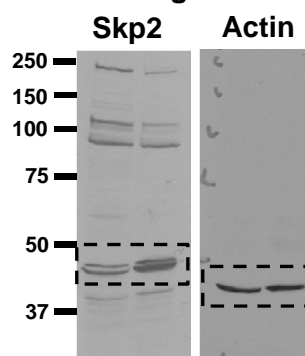

Supplement: Source Data for Figure 7 [file emboj2013243df7.pdf]

**Fig 8**

**Fig. 8D**

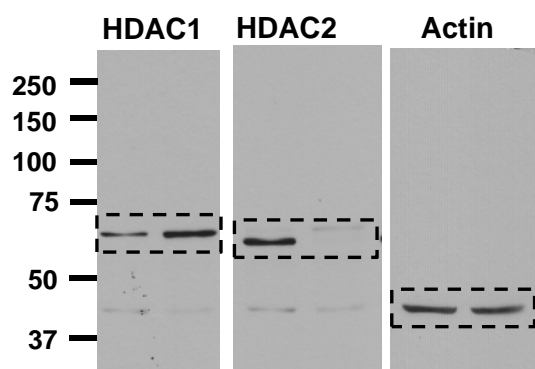

**Fig. 8G**

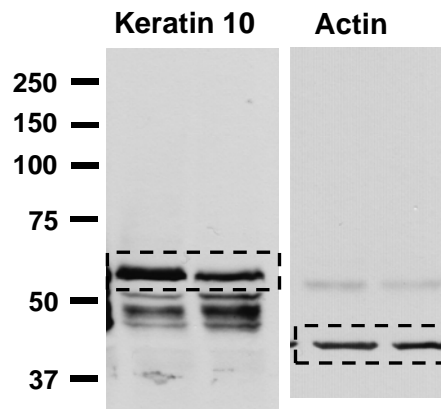

**Fig. 8I Input**

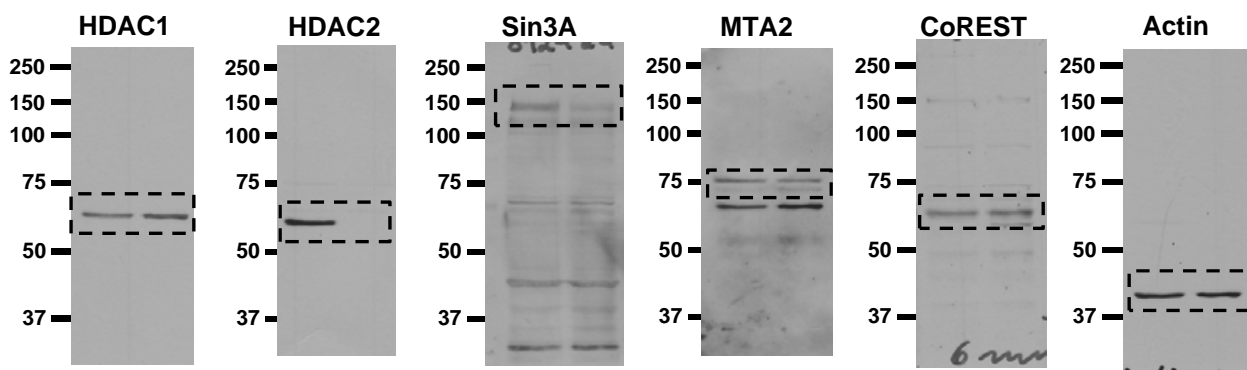

**Fig. 8I Sin3A-IP**

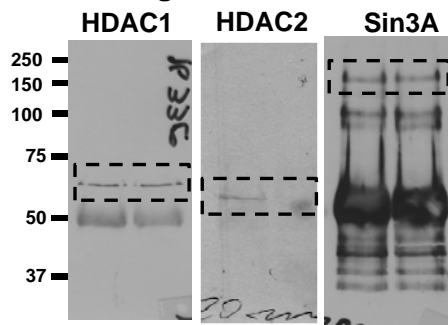

**Fig. 8I MTA2-IP**

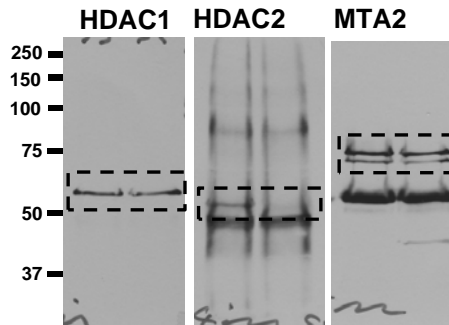

**Fig. 8I CoREST-IP**

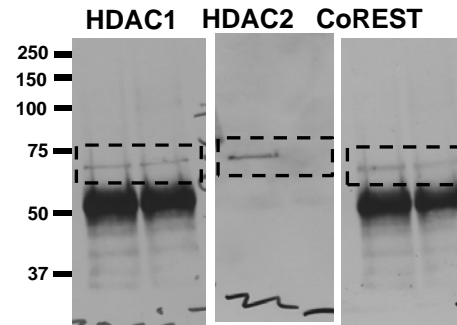

**Fig. 8K**

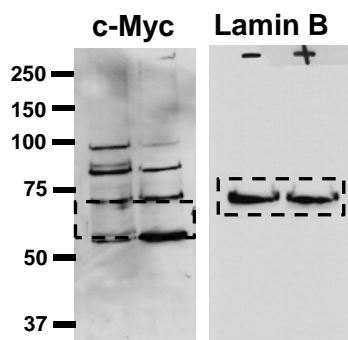

**Fig. 8K**

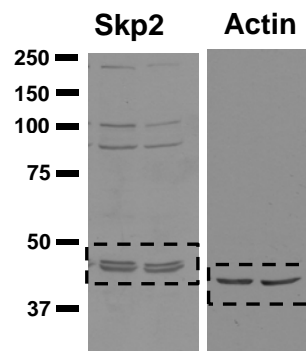

Supplement: Source Data for Figure 8 [file emboj2013243df8.pdf]
