# Supplementary material for: Divergent roles of HDAC1 and HDAC2 in the regulation of epidermal development and tumorigenesis
Source: EMBO J. 2013 Nov 15;32(24):3176–91. doi: 10.1038/emboj.2013.243 (PMC3981143; doi:10.1038/emboj.2013.243)

Fig 5

Fig. 5E Input

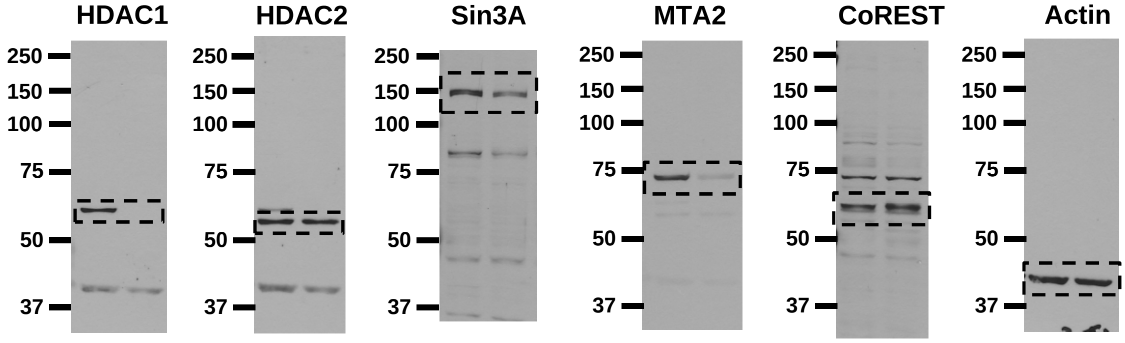

Fig. 5E Sin3A-IP

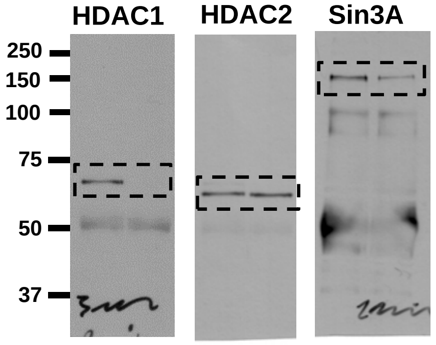

Fig. 5E MTA2-IP

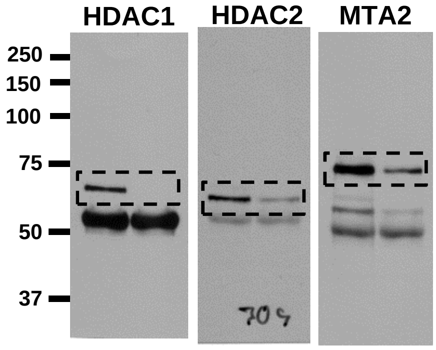

Fig. 5E CoREST-IP

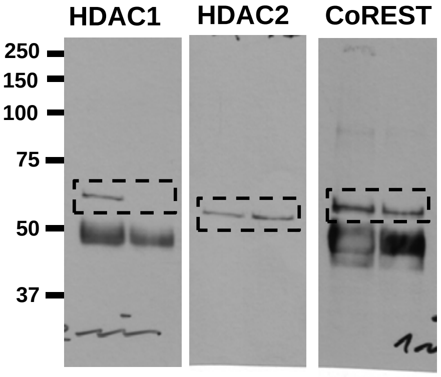

Fig. 5G Input

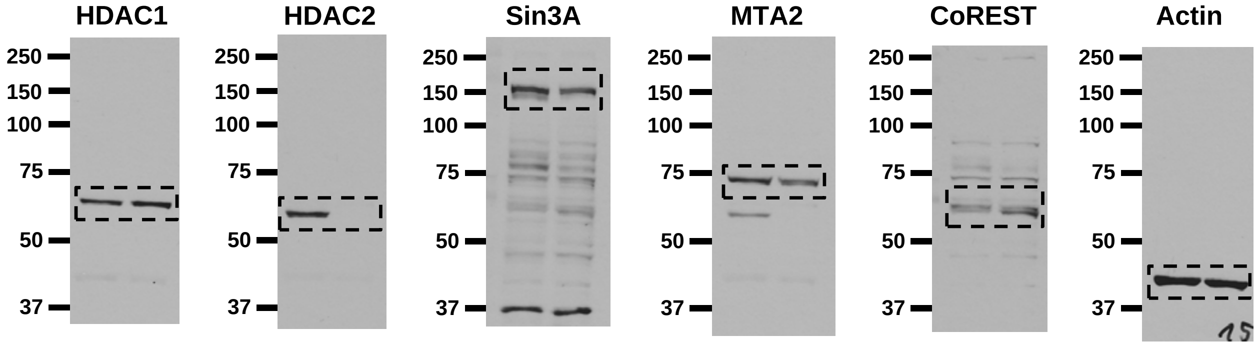

Fig. 5G Sin3A-IP

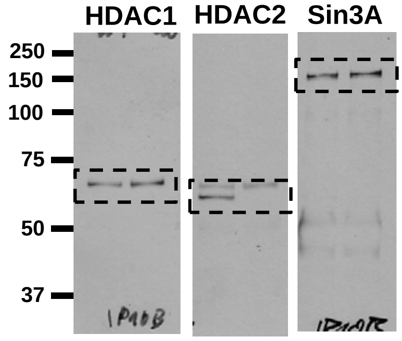

Fig. 5G MTA2-IP

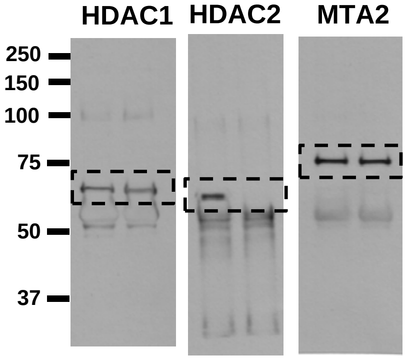

Fig. 5G CoREST-IP

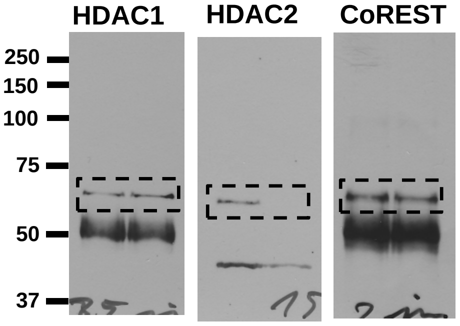

Supplement: Source Data for Figure 5 [file emboj2013243df5.pdf]
